# Immune–Inflammatory Biomarkers Predict Cognition and Social Functioning in Patients With Type 2 Diabetes Mellitus, Major Depressive Disorder, Bipolar Disorder, and Schizophrenia: A 1-Year Follow-Up Study

**DOI:** 10.3389/fneur.2022.883927

**Published:** 2022-06-02

**Authors:** Marta Garés-Caballer, Joan Vicent Sánchez-Ortí, Patricia Correa-Ghisays, Vicent Balanzá-Martínez, Gabriel Selva-Vera, Joan Vila-Francés, Rafael Magdalena-Benedito, Constanza San-Martin, Victor M. Victor, Irene Escribano-Lopez, Antonio Hernandez-Mijares, Juliana Vivas-Lalinde, Eduard Vieta, Juan C. Leza, Rafael Tabarés-Seisdedos

**Affiliations:** ^1^Teaching Unit of Psychiatry and Psychological Medicine, Department of Medicine, University of Valencia, Valencia, Spain; ^2^INCLIVA—Health Research Institute, Valencia, Spain; ^3^TMAP—Evaluation Unit of Personal Autonomy, Dependency and Serious Mental Disorders, University of Valencia, Valencia, Spain; ^4^Faculty of Psychology and Speech Therapy, University of Valencia, Valencia, Spain; ^5^Center for Biomedical Research in Mental Health Network (CIBERSAM), Institute of Health Carlos III, Madrid, Spain; ^6^Mental Health Unit of Catarroja, Valencia, Spain; ^7^IDAL—Intelligent Data Analysis Laboratory, University of Valencia, Valencia, Spain; ^8^Department of Physiotherapy, University of Valencia, Valencia, Spain; ^9^Service of Endocrinology and Nutrition, University Hospital Dr. Peset, Valencia, Spain; ^10^Foundation for the Promotion of Health and Biomedical Research in the Valencian Region (FISABIO), Valencia, Spain; ^11^Department of Physiology, University of Valencia, Valencia, Spain; ^12^Departament of Psychiatry, Mental Health Service of Manises, Valencia, Spain; ^13^Barcelona Bipolar and Depressive Disorders Unit, Institute of Neurosciences, Hospital Clínic of Barcelona, University of Barcelona, IDIBAPS, Catalonia, Spain; ^14^Department of Pharmacology and Toxicology, Faculty of Medicine, Complutense University of Madrid, Madrid, Spain

**Keywords:** immune–inflammation, executive function, social functioning, transdiagnostic analysis, diabetes mellitus, major depressive disorder, bipolar disorder, schizophrenia

## Abstract

**Background:**

Systemic, low-grade immune–inflammatory activity, together with social and neurocognitive performance deficits are a transdiagnostic trait of people suffering from type 2 diabetes mellitus (T2DM) and severe mental illnesses (SMIs), such as schizophrenia (SZ), major depressive disorder (MDD), and bipolar disorder (BD). We aimed to determine if immune–inflammatory mediators were significantly altered in people with SMIs or T2DM compared with healthy controls (HC) and whether these biomarkers could help predict their cognition and social functioning 1 year after assessment.

**Methods:**

We performed a prospective, 1-year follow-up cohort study with 165 participants at baseline (TB), including 30 with SZ, 42 with BD, 35 with MDD, 30 with T2DM, and 28 HC; and 125 at 1-year follow-up (TY), and determined executive domain (ED), global social functioning score (GSFS), and peripheral blood immune–inflammatory and oxidative stress biomarkers.

**Results:**

Participants with SMIs and T2DM showed increased peripheral levels of inflammatory markers, such as interleukin-10 (*p* < 0.01; η^2^*p* = 0.07) and tumor necrosis factor-α (*p* < 0.05; η^2^*p* = 0.08); and oxidative stress biomarkers, such as reactive oxygen species (ROS) (*p* < 0.05; η^2^*p* = 0.07) and mitochondrial ROS (*p* < 0.01; η^2^*p* = 0.08). The different combinations of the exposed biomarkers anticipated 46–57.3% of the total ED and 23.8–35.7% of GSFS for the participants with SMIs.

**Limitations:**

Participants' treatment, as usual, was continued without no specific interventions; thus, it was difficult to anticipate substantial changes related to the psychopharmacological pattern.

**Conclusion:**

People with SMIs show significantly increased levels of peripheral immune–inflammatory biomarkers, which may contribute to the neurocognitive and social deficits observed in SMIs, T2DM, and other diseases with systemic immune–inflammatory activation of chronic development. These parameters could help identify the subset of patients who could benefit from immune–inflammatory modulator strategies to ameliorate their functional outcomes.

## Introduction

For every individual, neurocognition and social abilities are essential for satisfactory daily living. Across chronic, somatic, and mental diseases, the impairment of these domains is a redundant and widely recognized finding (1, 2) and broadly conditions the day-to-day performance and social integration of people with such diagnoses. The highest percentage of disability-adjusted life years among severe mental illnesses (SMIs) has been reported for psychiatric conditions, such as major depressive disorder (MDD), schizophrenia (SZ), and bipolar disorder (BD) (3).

For SMIs, a pathophysiological role is suspected by immune–inflammatory mechanisms and a growing number of research groups have identified the patterns of neuroinflammation and immune dysfunction in a subset of patients with these conditions (4–7). Although the cause–effect relationship between an SMI development and immune–inflammatory processes still requires further elucidation (8, 9), certain observations support the hypothesis that the presence of immune–inflammatory alterations, is not a mere epiphenomenon. For instance, increased incidences of immune–inflammatory processes during the early life phases of these diseases demonstrate a bidirectional relationship among SMIs and autoimmune disorders (10, 11) that is also observed in first-degree relatives (12–14). Altered RNA transcripts of non-affected relatives of people with mood disorders (8, 15) may evince a disruption of the inflammatory system as a trait of abnormality in psychiatric disorders. Similarly, a disbalance in mechanisms controlling inflammation has been described in SMIs in the chronic and early stages (16, 17).

Both chronic mental and somatic diseases, such as type 2 diabetes mellitus (T2DM), may share a pro-inflammatory imbalance that might modulate brain functioning and lead to changes in mood, neurocognition, and behavior that are considered cardinal clinical manifestations of these entities (10, 18). In SMIs, as well as in T2DM, high levels of C-reactive protein (CRP), interleukin-6 (IL-6), tumor necrosis factor-alpha (TNF-α), and decreased IL-10 concentrations have been reported (19). These pro-inflammatory mediators induce insulin resistance by impairing intracellular signaling pathways and reducing the insulin-sensitizing effects of anti-inflammatory substances, such as IL-10. Chronically sustained, this alteration may account for the decreased neurogenesis and elevated neuronal death observed in diabetic brains. Multiple studies showed longitudinal associations of neurocognitive decline and dementia development with high concentrations of blood glucose due to insulin resistance (20–22). Moreover, mild-to-moderate neurocognitive impairment with reduced mental flexibility and psychomotor slowing has been redundantly identified in people with T2DM.

Misiak et al. (11) reported a transdiagnostic activation pattern for MDD, SZ, and BD consisting of increased peripheral levels of IL-6, soluble interleukin receptor-2 (sIL-R2), TNF-α, and IL-1-receptor antagonist (IL-1RA) in acutely relapsed patients. Additionally, an increased ratio of monocytes/lymphocytes was indicated for these SMIs (6) and high monocyte baseline counts seemed to relate to the subsequent need for long-term psychiatric care (23). Background inflammation in people with SMIs appears to be predictive of treatment response (24) and correlates with symptom severity (5, 7) and unfavorable long-term outcomes (13, 25). Peripheral and central inflammatory cytokines, circulating antibodies, altered levels of neurotrophic factors, imbalanced Th1/Th2-lymphocytic activity, and microglial activation seem to influence synaptic plasticity, neurotransmission, nuclear signal transduction, and neurogenesis (7, 26, 27). Thus, certain biomarkers of immune–inflammatory activity from peripheral blood may also partly underlie the detrimental effects of these entities on neurocognitive and social abilities (7, 11, 28).

Among the neurocognitive domains, executive functioning defines the effective coordination of simpler, interdependent processes which enable real-world adaptive success (29). According to this integration perspective, efficiently interconnected top–down executive processes, such as cognitive flexibility, verbal fluency, and working memory, constitute a multimodal system of execution that optimizes cognitive resource employment to achieve flexible and adaptive behavior. As a transdiagnostic observation, altered executive performance is a phenomenon converging chronic disorders with different central nervous system etiologies (30).

Epidemiologically, SMIs have a bidirectional association with cardiometabolic diseases (2, 31), which have a high heritability rate and the well-known trait of hyperactive inflammatory function (32). Certain research groups have delved into the potential benefits of add-on therapies with anti-inflammatory and/or antioxidant interventions and positive effects on symptom scores have been reported for MDD, SZ, and BD (33–36), although the results are still inconclusive (37). In this context, a genetic overlap between cardiometabolic diseases and SMIs is suspected and pathways regulating the hypothalamic–pituitary–adrenal axis, glucose metabolism, neurogenesis, and other homeostatic systems have been suggested as transdiagnostic mechanisms underlying these pathologies (28, 32, 38). A better understanding of the interaction between immune–inflammatory activity and brain function could elucidate a novel, individualized approach to cognition and social ability impairments, with transdiagnostic repercussions for the management of both somatic and mental diseases. In this context, we aimed to determine if peripheral biomarkers of immune–inflammatory activity could help to predict cognition and social functioning in people with SMIs and T2DM from a transdiagnostic perspective by implementing a follow-up study design.

## Materials and Methods

### Study Design and Ethical Considerations

This study shows partial results of a more extensive study that seeks to identify and validate peripheral biomarkers for neurocognitive deficits in MDD, BD, SZ, and T2DM. Only variables that could provide clarity to the aim of the study were included in the analyses. This prospective, comparative cohort study was conducted between April 2015 and January 2018. Several biomarkers, clinical, sociodemographic data, neurocognitive performance, and social functioning data were collected at baseline (TB) and after 1 year (TY). Individuals with SMI were recruited from the mental health units in several towns in the province of Valencia, Spain (Foios, Catarroja, Paterna, and Sagunto), the Psychiatry Outpatient Clinic and Endocrinology Department of the University Hospital Dr. Peset and the Mental Health Unit of the Health Center of Miguel Servet, in Valencia City, Spain. The healthy controls (HCs) resided in the same geographical areas and as much as possible were matched in age, sex, socio–economical status, and years of education. The study procedures were explained to the participants, and all participants provided informed consent. The study was conducted following the ethical principles of the Declaration of Helsinki and approved by the ethical committees of the University of València Clinic Hospital, University Hospital Dr. Peset, and University and Polytechnic La Fe Hospital.

### Participants

The study began (TB) with a cohort consisting of 165 participants as detailed in the following: Total 30 patients with SZ, 42 with type-I BD, 35 with MDD, 30 with T2DM, and 28 non-relative individuals who were genetically unrelated HC. At TY, data from 125 participants were collected: Total 27 patients with SZ, 29 with type-I BD, 25 with MDD, 25 with T2DM, and 19 HC. The diagnoses of SZ, BD, and MDD were established following the criteria of the *Diagnostic and Statistical Manual of Mental Disorders*, Fifth Edition (39). The T2DM diagnosis was based on the *American Diabetes Association Standards of Medical Care in Diabetes* (40). For all groups the same exclusion criteria were applied at TB and TY: clinical conditions that hindered the study design, current hospitalization, documentation of neurocognitive impairment (intellectual disability or dementia), disability or inability that prevented understanding the protocol, current substance use disorder, pregnancy, steroids, corticosteroids, antioxidants or antibiotics intake, immunologic therapies, fever over 38°C, and history of vaccination within 4 weeks of the evaluation. Patients diagnosed with MDD and BD had to be clinically stable without presenting an acute affective episode and only individuals with SZ who were in a clinically stable state were included. Patients with T2DM with severe diabetic neuropathy and kidney disease (serum creatinine > 1.5 mg/dL) were discarded. The absence of physical illness, pharmacological treatments, and family history of SMI in first-degree relatives were mandatory for an HC to be recruited. All selected participants had the ability to understand and provided written consent.

### Clinical and Neuropsychological Assessments

Experienced psychologists and psychiatrists, and members of the research group conducted clinical and neuropsychological assessments. At TB, the general sociodemographic data, including sex, age, and years of education, were collected; for the individuals with a diagnosis, age of disease onset and illness duration were also obtained.

Clinical evaluations were conducted using the following scales: Hamilton Depression Rating Scale (41); Young Mania Rating Scale (42); Positive and Negative Symptoms Scale (43); and Clinical Global Impression Scale (44).

Social functioning was evaluated using the Functional Assessment Short Test (45); Short Form 36 Health Survey Questionnaire (46); and the World Health Organization Quality of Life abbreviated version WHOQOL-BREF (47). A global social functioning score (GSFS) was calculated by averaging the three-scale total scores. The significant relationships between test scores were previously checked in the whole sample, at baseline and at 1-year follow-up, to reduce possible biases due to different functional measures.

The neurocognitive performance was evaluated using a battery of cognitive tests and subtests previously used by our group (48–55). Test and subtests scores were grouped into the following four executive functions: (1) Cognitive flexibility: Stroop Color and Word Test (SCWT), Color/Word Subtest (56), and Wisconsin Card Sorting Test Categories Completed and Perseverative Errors scored (57); (2) verbal fluency: Verbal Fluency Tasks Semantic and Phonemic Forms (58); (3) working memory: Trail Making Test (TMT) Part B (59), and Wechsler Adult Intelligence Scale Third Edition (WAIS-III) Digit Span-B Subtest (60); and (4) processing speed: Finger Tapping Test (59, 61), WAIS-III Digit Symbol Coding Subtest (60), SCWT Color and Word Subtests (56), and TMT Part A (59). Executive domain (ED) was calculated by averaging the four executive functions' total scores.

### Determination of Biomarkers in Peripheral Blood

Venous blood extraction was performed, and the serum and plasma samples were kept in a freezer at −80°C. (1) Cytokines: Serum cytokine concentration was determined using Luminex® X-MAP technology (Luminex Corp., Austin, TX, USA) based on flow cytometry. The following cytokines were analyzed: IL-6, interleukin 10 (IL-10), and TNF-α. Sample processing and data analysis were performed according to the manufacturer's instructions. (2) Oxidative stress markers and mitochondrial metabolism: Oxidative stress in leukocytes was evaluated using fluorimetry techniques in a fluoroscan (Synergy MX). Plated in 96-well plates, 100,000 cells in each, were incubated 30 min at 37°C with the corresponding fluorochrome: dichlorofluorescein diacetate indicated reactive oxygen species (ROS) production (485-nm excitation, 535-nm emission), MitoSOX measured mitochondrial ROS (mROS) (510-nm excitation, 580-nm emission), tetramethylrodamin methyl ester (552-nm excitation, 574-nm emission) assessed membrane potential, nonylacridin orange mitochondrial mass (495-nm excitation, 519 nm emission), and 5-chloromethylfluorescein diacetate measured intracellular glutathione (492-nm excitation, 517 nm-emission). We used the monocyte cell line U-937 as an internal control to avoid the possible fluctuation of fluorescence associated with time. Serum lipid peroxidation levels were measured using a commercial thiobarbituric acid reactive substances kit and performed according to the manufacturer's instructions. The CRP levels were determined by an immunonephelometric assay (Behring Nephelometer II, Dade Behring, Inc., Newark, DE, USA).

### Statistical Analyses

Data were analyzed using Statistical Package for Social Sciences (SPSS), version 26.0 for Windows (62). The descriptive analyses were conducted using a one-way analysis of variance (ANOVA) for continuous variables and the Chi-squared test for categorical variables. Normality was assumed for all continuous variables because the sample was sufficiently representative of the target population and was statistically verified. This fact guaranteed that the variable groups for TB and TY could be assessed using a one-way analysis of covariance (ANCOVA), with sex, age, years of education, and BMI as covariates. A *post hoc* analysis with a Bonferroni corrected pairwise *t*-test was performed to examine the differences between groups. The direct scores obtained for executive performance and social functioning were transformed into *Z*-scores. For the calculation of the *Z*-scores, the mean and standard deviation (SD) of the HC at TB were taken as reference values. To test the predictive capacity of biomarkers at TB to explain the variance in executive performance and social functioning at TY, a linear regression analysis was performed using a predictive model that tested all biomarkers in each group. For all analyses, *p* < 0.05 was considered statistically significant. The procedure to create the predictive models was as follows: First, a predictive analysis was performed with biomarkers one by one, then predictive models were generated including and combining the more statistically powerful biomarkers; therefore, we obtained the optimal predictive combination. No more than five variables were included in each model, thus ensuring the correct performance of the analysis.

## Results

### Sample Description

The sociodemographic and clinical data of the participants at TB are shown in Table 1. At TB, individuals with SMIs or T2DM represented 83%, and HC 17% of the total sample. Females accounted for 48% of the total participants. The mean age of the whole sample was 46.4 (SD: 12.9) years. The mean years of education of all participants were 12.5 (SD: 4.7), similar among the clinical groups and significantly different compared to HC. Depressive symptoms were significantly higher in the individuals with SMI compared to HC; among them, the participants with MDD showed the most accentuated scores. Individuals with BD showed significantly high maniac symptoms compared to the HC group. Patients with SZ showed increased psychotic symptoms compared to the other groups. Significant differences were also found for participants with SZ, BD, MDD, and T2DM in terms of clinical severity, age of onset, and illness duration; individuals with SZ had the worst clinical pattern, those with T2DM had the latest age of onset, and those with BD had the longest illness duration. At TY, the attrition rate was 24.3 because 40 participants were lost to follow-up.

**Table 1:** Sociodemographic and clinical characteristics of the sample at T1.

**Variables^a^**	**HC**	**T2DM**	**MDD**	**BD**	**SZ**	**Statistical analyses**	
	**(*n* = 28)**	***(n* = 30)**	**(*n* = 35)**	**(*n* = 42)**	**(*n* = 30)**	**F(*p*)^e^**	***Post hoc* test^g^**
**Sociodemographic**							
Sex^b^^,^ ^f^^,^ ^h^	18(64%)	9(30%)	24(68%)	21(50%)	7(23%)	20.1^****^	SZ,T2DM < HC,MDD SZ < BD
Age	36.6(14.5)	57.3(9.3)	47.3(11.8)	50.0(9.5)	40.8(10.7)	15.3^****^	HC < MDD,BD,T2DM SZ,MDD < T2DM SZ < BD
Years of education	16.1(3.3)	12.5(5.8)	11.9(4.3)	11.6(4.4)	10.4(3.3)	7.1^****^	SZ,BD,MDD,T2DM < HC
**Clinical**							
BMI	24.9(5.1)	30.4(4.3)	28.6(5.8)	29.7(5.7)	31.9(5.4)	6.9^****^	HC < BD,T2DM,SZ
HDRS^c^	2.0(1.8)	3.9(3.9)	11.6(8.3)	6.4(4.4)	7.0(5.8)	14.2^****^	HC < BD,SZ,MDD T2DM,BD,SZ < MDD
YMRS^c^	0.8(1.6)	1.5(2.2)	1.9(2.6)	3.5(4.5)	3.2(4.9)	3.4^**^	HC < BD
PANSS-P^c^	7.0(0.0)	7.0(0.0)	7.0(0.3)	8.5(3.8)	10.6(4.3)	10.6^****^	HC,T2DM,MDD,BD < SZ
PANSS-N^c^	7.0(0.0)	7.1(0.7)	8.4(4.9)	10.3(6.5)	18.6(10.1)	20.1^****^	HC,T2DM,MDD,BD < SZ
PANSS-G^c^	16.0(0.0)	17.0(2.3)	19.8(8.6)	22.7(9.9)	31.8(12.7)	16.9^****^	HC,T2DM,MDD,BD < SZ HC < BD
CGI^c^	–	1.9(1.0)	3.3(1.2)	3.5(0.7)	4.5(1.0)	31.3^****^	T2DM,MDD,BD < SZ T2DM < MDD,BD
Age of onset^d^	–	44.3(9.8)	35.3(12.1)	26.5(8.6)	25.6(8.0)	25.6^****^	SZ,BD < MDD,T2DM MDD < T2DM
Illness duration^d^	–	13.0(9.0)	12.0(11.6)	23.4(11.5)	15.2(8.4)	9.6^****^	MDD,T2DM,SZ < BD

a *Expressed as mean (SD) except when indicated*,

b *Female n (%)*.

c *Lower scores represent a better outcome*.

d *Years*.

e *ANOVA*.

f *Chi-squared test*.

g *Bonferroni test*.

h *Mann–Whitney U test. T1, Time 1; HC, Healthy Control; T2DM, Type-2 Diabetes Mellitus; MDD, Major Depressive Disorder; BD, Bipolar Disorder; SZ, Schizophrenia; BMI, Body Mass Index; HDRS, Hamilton Rating Scale for Depression; YMRS, Young Mania Rating Scale; PANSS, Positive and Negative Syndrome Scale; P, Positive; N, Negative; G, General; CGI, Clinical Global Impression. ANOVA, Analysis of variance. NS, Not Significant*.

(** *p ≤ 0.01*;

**** *p ≤ 0.0001)*.

### Between-Group Comparisons of Immune–Inflammatory Biomarkers

The results registered in Table 2 showed significant differences between individuals with T2DM compared to individuals with MDD and SZ for IL-10 at TB (*p* < 0.0001; η^2^*p* = 0.14), as well as between the those with HC and SZ for TNF-α at TY (*p* < 0.05; η^2^*p* = 0.08); individuals with SZ obtained significantly higher scores for both biomarkers.

**Table 2:** Biomarkers at T1 and T2.

	**HC**		**T2DM**		**MDD**		**BD**		**SZ**			**Statistical analyses**				
**Variables^a^**	**T1 (*n* = 28)**	**T2 (*n* = 19)**	**T1 (*(n* = 30)**	**T2 (*n* = 25)**	**T1 (*n* = 35)**	**T2 (*n* = 25)**	**T1 (*n* = 42)**	**T2 (*n* =2 9)**	**T1 (*n* = 30)**	**T2 (*n* = 27)**	**T1 (*p*)^b^**	***Post hoc* test^c^**	**η^2^*p*^d^**	**T2 (*p*)^b^**	***Post hoc* test^c^**	**η^2^*p*^d^**
**Neuroinflammatory markers**																
IL-6	2.3(0.9)	2.9(1.7)	2.6(1.3)	3.6(5.3)	2.7(2.8)	3.0(1.5)	3.6(3.2)	3.1(2.8)	2.6(1.6)	5.1(7.2)	NS			NS		
IL-10	50.3(19.7)	34.3(9.7)	13.7(4.4)	28.4(7.4)	35.2(31.4)	7.3(8.9)	21.0(21.6)	12.1(15.4)	52.5(45.4)	17.7(7.5)	6.5^****^	T2DM < MDD,SZ BD < SZ	0.14	NS		
TNF-α	8.4(1.8)	9.4(2.9)	9.1(1.8)	10.1(2.9)	9.4(3.0)	10.6(3.2)	10.7(3.8)	11.1(3.0)	9.3(3.1)	13.4(6.7)	NS			2.6^*^	HC < SZ	0.08
PCR-us	3.1(3.6)	1.9(1.4)	3.8(6.5)	4.7(7.8)	3.6(4.4)	3.7(3.3)	3.3(2.8)	3.0(3.4)	4.7(5.2)	5.2(5.8)	NS			NS		
**Oxidative stress markers**																
GSH	230.0(126.6)	188.6(75.2)	190.3(86.0)	176.3(87.0)	196.9(73.7)	187.1(96.5)	204.8(91.5)	199.9(90.5)	200.7(96.5)	193.1(97.5)	NS			NS		
ROS	131.1(51.8)	356.4(162.0)	114.3(15.3)	239.1(118.4)	179.1(121.6)	318.1(174.2)	134.3(83.8)	294.9(158.2)	133.1(78.7)	344.4(229.3)	3.2^**^	HC < MDD	0.07	NS		
mROS	181.7(54.2)	203.6(67.8)	172.8(55.0)	191.0(91.8)	148.7(60.6)	208.2(98.3)	191.2(77.5)	240.0(124.2)	144.8(46.8)	196.8(109.7)	NS			NS		
SOD	107.0(38.3)	107.0(40.1)	93.6(17.4)	95.7(16.3)	98.3(18.9)	99.6(22.0)	118.0(59.7)	97.8(20.3)	101.9(17.9)	102.3(37.6)	NS			NS		
**Hemogram**																
RBC	4.5(0.4)	4.7(0.4)	4.5(0.4)	4.6(0.3)	4.7(0.3)	4.8(0.3)	4.5(0.4)	4.6(0.3)	4.6(0.3)	4.6(0.3)	NS			NS		
HGB	13.4(1.4)	13.6(2.0)	13.7(1.1)	13.5(1.1)	13.9(1.0)	13.9(1.4)	13.9(1.3)	13.9(1.1)	13.9(1.5)	13.9(1.4)	NS			NS		
HCT	40.4(3.9)	41.3(5.2)	41.2(3.6)	41.6(3.6)	42.4(3.2)	42.6(3.7)	42.4(3.9)	42.3(3.4)	42.6(4.1)	42.4(3.8)	NS			NS		
PLT	240.8(64.4)	261.6(94.6)	219.4(53.3)	233.6(48.8)	241.2(67.1)	237.7(74.1)	244.9(58.0)	237.8(50.3)	247.4(64.1)	254.2(61.0)	2.6*	T2DM < SZ	0.06	NS		
WBC	7.5(2.6)	7.6(3.1)	7.4(1.5)	7.1(1.3)	7.4(1.8)	7.8(1.9)	7.6(2.0)	7.7(1.9)	7.6(2.2)	7.6(2.4)	NS			NS		
WBC-N	56.8(8.4)	55.8(10.0)	57.0(8.3)	59.7(8.9)	57.4(8.1)	53.0(13.2)	59.7(8.7)	57.4(8.3)	57.8(7.2)	57.9(8.1)	NS			NS		
WBC-AN	4.3(2.1)	4.4(2.5)	4.3(1.3)	4.3(0.9)	4.2(1.2)	4.2(1.7)	4.7(1.8)	4.4(1.3)	4.4(1.6)	4.5(1.8)	NS			NS		
WBC-L	32.4(7.8)	32.9(9.6)	32.5(7.4)	29.1(8.6)	32.5(7.4)	35.6(11.9)	29.5(8.5)	31.1(7.4)	31.6(7.2)	31.5(7.2)	NS			NS		
WBC-AL	2.3(0.6)	2.4(0.8)	2.3(0.6)	2.1(0.6)	2.3(0.7)	2.6(0.8)	2.1(0.5)	2.3(0.8)	2.3(0.7)	2.3(0.7)	NS			NS		
WBC-M	7.7(2.2)	7.9(2.4)	7.6(1.8)	7.8(1.6)	8.3(5.9)	7.8(1.9)	7.4(1.8)	7.5(1.4)	7.2(1.4)	7.6(1.6)	NS			NS		
WBC-AM	0.5(0.1)	0.5(0.2)	0.5(0.1)	0.5(0.1)	0.5(0.1)	0.6(0.2)	0.5(0.1)	0.5(0.1)	0.5(0.1)	0.5(0.1)	NS			NS		

a *Expressed as mean (SD)*.

b *ANCOVA*.

c *Bonferroni test*.

d *Partial Eta-Squared (η^2^p). T1, Time 1; T2, Time 2; HC, Healthy Control; T2DM, Type-2 Diabetes Mellitus; MDD, Mayor Depressive Disorder; BD, Bipolar Disorder; SZ, Schizophrenia; IL-6, Interleukin-6; IL-10, Interleukin-10; TNF-α, Tumor Necrosis Factor alpha; PCR-us, Ultra-sensitive Protein C; GSH, Glutathione; ROS, Reactive Oxygen Species; mROS, Mitochondrial Reactive Oxygen Species; SOD, Superoxide Dismutase; RBC, Red Blood Cells; HGB, Hemoglobin; HCT, Hematocrit; PLT, Blood Platelets; WBC, White Blood Cell; N, Neutrophils; AN, Absolute Neutrophils; L, Lymphocytes; AL, Absolute Lymphocytes; M, Monocytes; AM, Absolute Monocytes; NS, Not Significant. (NS = p > 0.05*;

* *p ≤ 0.05*;

** *p ≤ 0.01*;

**** *p ≤ 0.0001). Effect size (η^2^p: small ≈ 0.02; moderate ≈ 0.15; large ≈ 0.35)*.

Similarly, we found significant differences for several oxidative stress markers; participants with MDD showed significant differences compared to those with HC for ROS (*p* < 0.01; η^2^*p* = 0.07). Participants with MDD obtained significantly higher scores for biomarkers.

However, those with T2DM showed significant differences compared to those with SZ for platelets (*p* < 0.05; η^2^*p* = 0.06). For all cases, the effect size was from small-to-moderate. The differences between the time points within each group were not significant.

### Between-Group Comparison of Executive and Social Functioning

The executive and social functioning at TB and TY of the five groups of participants, respectively, are shown in Table 3. In general terms, the results obtained indicated that individuals with BD and SZ had worse executive functioning compared to the other groups, those with SZ were the most impaired (*p* < 0.0001; η^2^*p* = 0.15). Moreover, individuals with MDD and T2DM submitted to an attenuated processing speed, but not as severe as those with BD or SZ (*p* < 0.0001; η^2^*p* = 0.18), while the HCs demonstrated optimal executive performance compared to the clinical groups (*p* < 0.001; η^2^*p* = 0.07 to 0.18). In the same way, the results of social functioning indicated that participants with SMI had worse social functioning compared with those with T2DM and the HCs (*p* < 0.0001; η^2^*p* = 0.37). These findings were maintained at TY (*p* < 0.0001; η^2^*p* = 0.08 to 0.34). The moderate-to-large effect sizes were observed at both assessments. The within-group executive and social functioning over time did not significantly differ.

**Table 3:** Executive functions and social functioning at T1 and T2.

	**HC**		**T2DM**		**MDD**		**BD**		**SZ**			**Statistical analyses**				
**Variables^a^**	**T1 (*n* = 28)**	**T2 (*n* = 19)**	**T1 (*n* = 30)**	**T2 (*n* = 25)**	**T1 (*n* = 35)**	**T2 (*n* = 25)**	**T1 (*n* = 42)**	**T2 (*n* =2 9)**	**T1 (*n* = 30)**	**T2 (*n* = 27)**	**T1 (*p*)^b^**	***Post hoc* test^c^**	**η^2^*p*^d^**	**T2 (*p*)^b^**	***Post hoc* test^c^**	**η^2^*p*^d^**
***Executive functions***																
**CF**	0.0(0.7)	0.2(0.8)	−1.0(1.1)	−1.2(1.2)	−1.0(1.3)	−0.7(1.3)	−1.4(1.2)	−1.7(1.4)	−1.9(1.5)	−1.7(1.5)	4.5^***^	SZ < MDD, T2DM, HC	0.10	4.8^***^	SZ < T2DM,MDD,HC	0.14
**VF**	0.0(0.8)	0.2(0.9)	−0.4(1.0)	−0.3(0.9)	−0.3(0.9)	−0.2(0.9)	−0.5(0.9)	−0.4(0.8)	−1.2(0.7)	−1.0(0.7)	3.1^**^	SZ < MDD	0.07	2.7^*^	SZ < MDD	0.08
**WM**	0.0(0.8)	0.1(0.7)	−1.3(1.5)	−1.4(1.5)	−0.8(1.2)	−1.1(1.2)	−2.2(2.6)	−1.3(1.9)	−2.4(1.8)	−2.3(2.0)	5.4^****^	SZ,BD < MDD	0.12	4.3^**^	SZ < BD,T2DM,MDD,HC	0.13
**PS**	0.0(0.6)	0.2(0.5)	−1.1(1.1)	−1.2(1.0)	−1.1(1.0)	−1.2(0.9)	−1.7(1.3)	−1.4(1.0)	−1.9(1.2)	−1.9(1.2)	8.6^****^	SZ,BD < T2DM,HC SZ < MDD	0.18	9.5^****^	SZ,BD,MDD < HC SZ < BD, MDD, T2DM	0.24
***Executive domain***																
**ED**	0.0(0.6)	0.2(0.5)	−1.0(1.0)	−1.0(1.0)	−0.8(0.9)	−0.8(0.9)	−1.5(1.3)	−1.2(1.1)	−1.9(1.1)	−1.7(1.2)	7.2^****^	SZ < T2DM,MDD,HC	0.15	7.0^****^	SZ < BD,T2DM,MDD,HC	0.19
***Social functioning***																
**GSFS**	0.0(0.7)	0.0(0.8)	−1.0(1.3)	−1.4(1.8)	−3.2(1.7)	−4.5(2.9)	−2.8(1.3)	−3.9(1.7)	−2.9(1.4)	−3.8(2.1)	23.1^****^	MDD,SZ,BD < T2DM, HC	0.37	14.8^****^	MDD,SZ,BD < T2DM, HC	0.34

a * Z-Scores expressed as mean(SD)*.

b *ANCOVA*.

c *Bonferroni test*.

d *Partial Eta-Squared (η^2^p). T1, Time 1; T2, Time 2; HC, Healthy Control; T2DM, Diabetes Mellitus Type 2; MDD, Mayor Depressive Disorder; BD, Bipolar Disorder; SZ, Schizophrenia; CF, Cognitive Flexibility; VF, Verbal Fluency; WM, Working Memory; PS, Processing Speed; ED, Executive Domain; GSFS, Global Social Functioning Score; NS, Not Significant. (NS = p > 0.05*;

* *p ≤ 0.05*;

** *p ≤ 0.01*;

*** *p ≤ 0.001*;

**** *p ≤ 0.0001)*.

*Effect size (η^2^p: small ≈ 0.02; moderate ≈ 0.15; large ≈ 0.35)*.

### Predictive Power of Immune–Inflammatory Biomarkers of Executive Performance and Social Functioning

The results of the relative contributions of the immune–inflammatory biomarkers studied at TB, to explain the variation in executive performance and social functioning scores at TY are shown in Supplementary Tables S1–S5.

**Figure 1:**
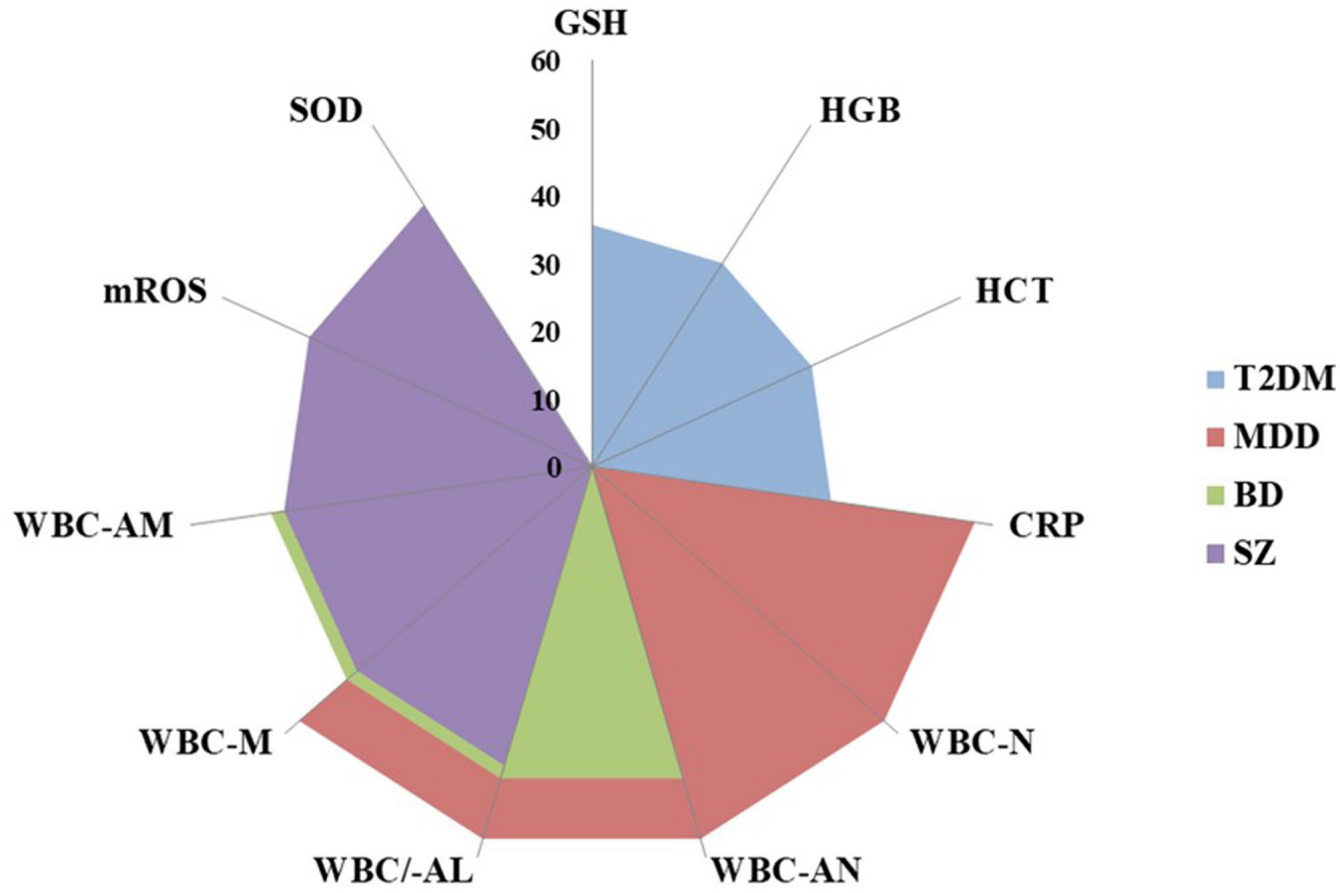
Shared immune-inflammatory biomarkers that predict neurocognitive functioning in clinical groups. T2DM, type 2 diabetes mellitus; MDD, major depressive disorder; BD, bipolar disorder; SZ, schizophrenia; GSH, glutathione; HGB, hemoglobin; HCT, hematocrit; CRP, C-reactive protein; WBC, white blood cells; N, neutrophils; AN, absolute neutrophils; AL, absolute lymphocytes; M, monocytes; AM, absolute monocytes; mROS, mitochondrial reactive oxygen species; SOD, superoxide dismutase.

Similarly, the different and significant combinations of immune–inflammatory biomarkers explaining a large proportion of executive and social functioning variance at TY were found. In participants with T2DM, baseline GSH constituted a key biomarker for the prediction of executive and social functioning, in combination with pro-inflammatory CRP, red blood cell activity [hemoglobin (HGB) and hematocrit (HCT)], and with oxidative stress biomarkers [ROS and superoxide dismutase (SOD)] between 25.9 and 38.6% of the variance at TY was explained. It should be noted that IL-6 alone predicted verbal fluency, explaining 23.5% variance at TY. Regarding MDD, between 32.7% and 57.3% of the executive functioning variance at TY was explained by leukocyte [white blood cell-neutrophils (WBC-N), -absolute neutrophils (WBC-AN), and -monocytes (WBC-M)] and pro-inflammatory activity (IL-6, IL-10, TNF-α, and CRP) together with oxidative stress biomarkers (ROS and SOD). Moreover, IL-6 alone predicted cognitive flexibility, processing speed and executive domain, explaining between 33.2 and 39.8% of variance at TY. Likewise, WBC and red blood cell (RBC) activities (HGB and WBC-AN) were considered significant biomarkers to predict social functioning, explaining 23.8% of variance. In participants with BD, 26.4–49.8% of executive functioning variance at TY was explained by a combination of pro-inflammatory factors (IL-6 and CRP), RBC and WBC activities [HGB; HCT; and WBC-N, -AN, -lymphocytes (L), -absolute lymphocytes (AL), -M, -AM] together with oxidative stress biomarkers (GSH and SOD). The combination of pro-inflammatory (TNF-α) and leukocyte activity (WBC-L), together with oxidative stress (ROS) were significant and explained 24.7% of the social functioning variance. In terms of those with SZ, 13.8–46.0% of executive functioning and social functioning variance at TY was explained by leukocyte (WBC-M and -AM) and pro-inflammatory activity (IL-6, IL-10, and CRP) together with oxidative stress biomarkers (ROS, mROS, and SOD). Moreover, IL-6 (13.8%) and CRP (16.4%), solely predicted cognitive flexibility and working memory, respectively. Across the clinical groups, shared immune–inflammatory biomarkers were found to predict executive functioning at TY (Figure 1).

**Figure 2:**
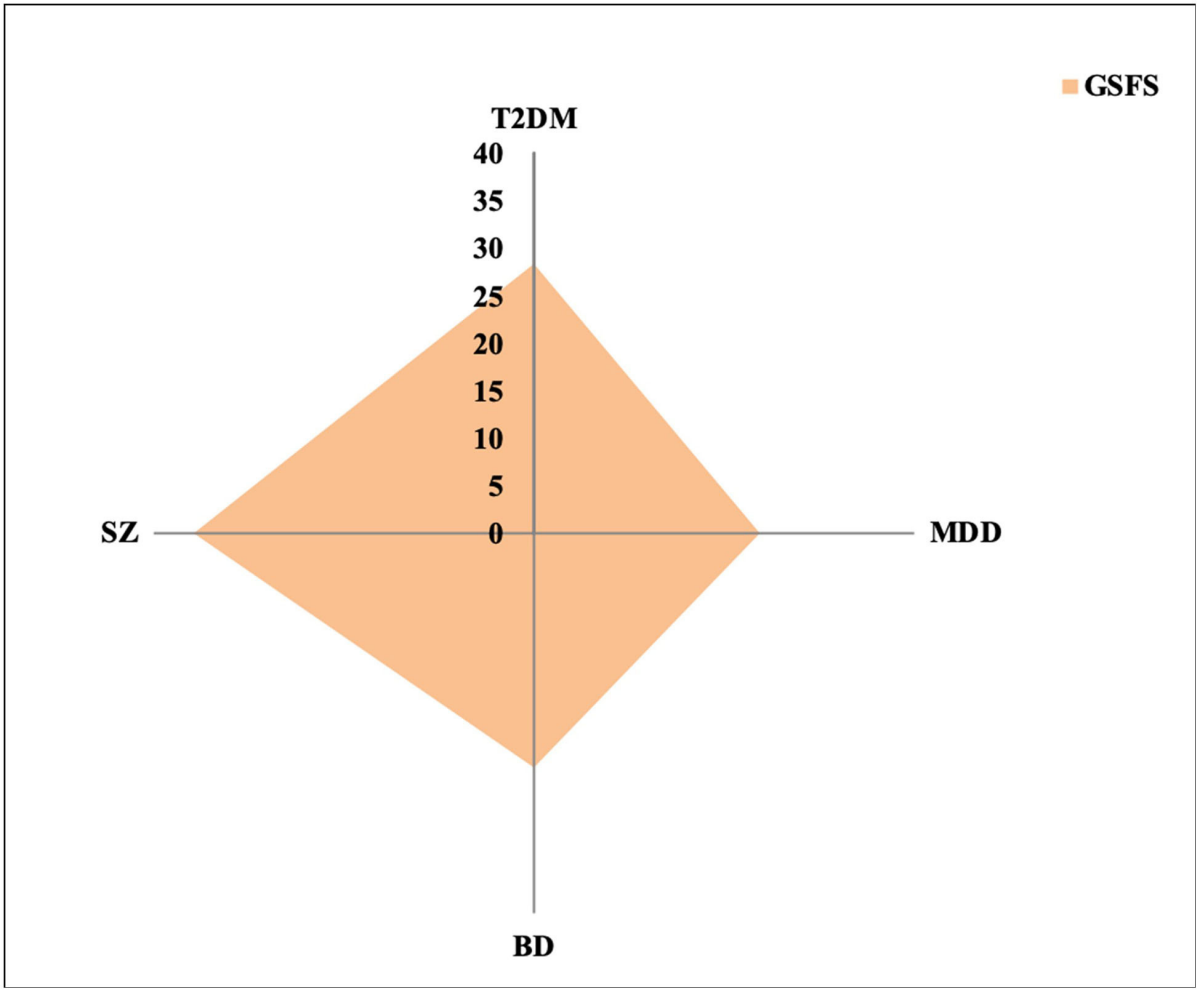
Amount of GSFS variance explained by immune-inflammatory biomarkers across clinical groups. GSFS, Global Social Functioning Score; T2DM, type 2 diabetes mellitus; MDD, major depressive disorder; BD, bipolar disorder; SZ, schizophrenia.

Similarly, inflammatory and oxidative stress molecules were common key factors in predicting social functioning at TY, being more robust in the SZ group (Figure 2).

**Figure 3:**
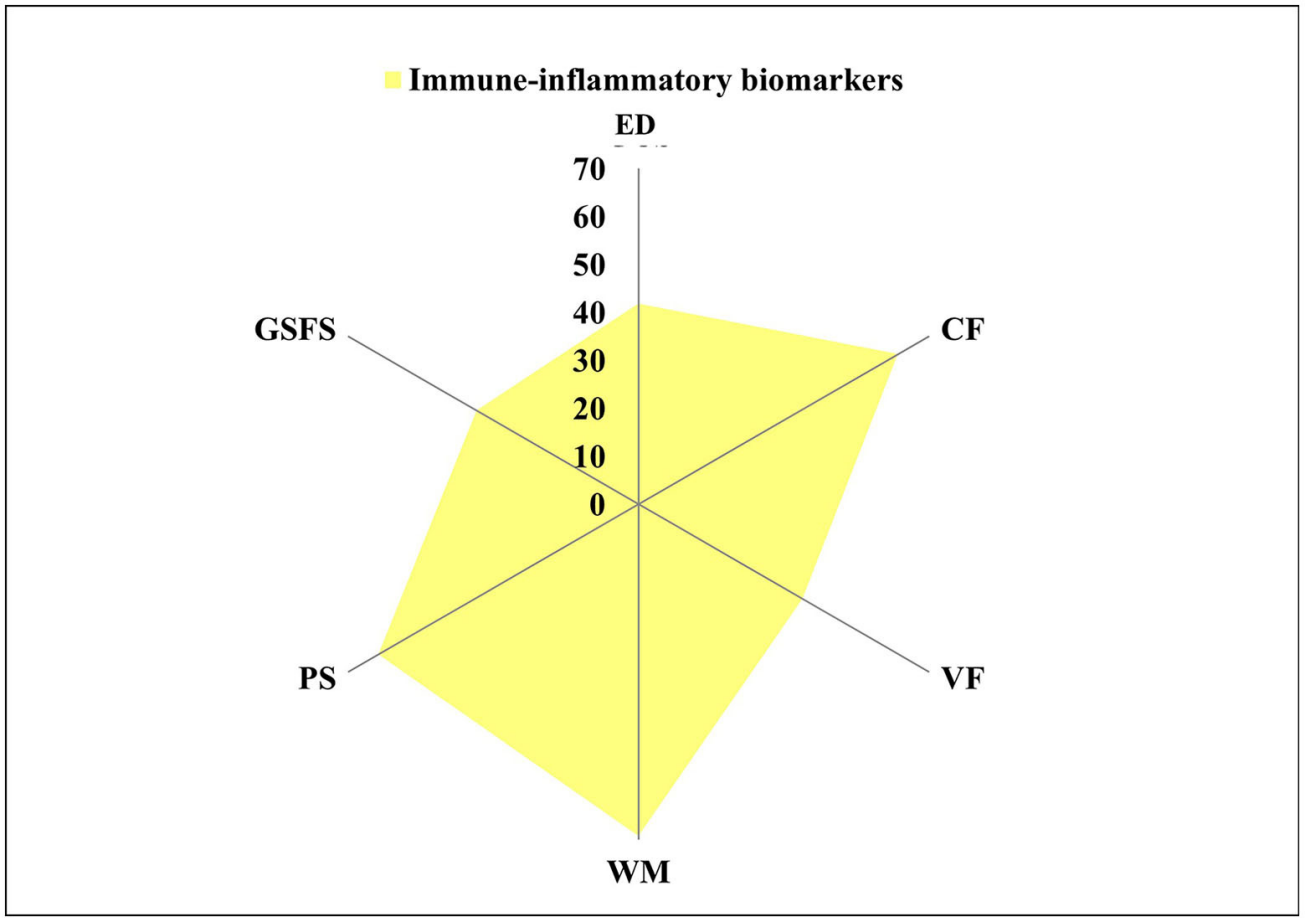
Variance explained by immune-inflammatory biomarkers in HC group. HC, healthy control; ED, Executive Domain; CF, cognitive flexibility; VF, verbal fluency; WM, working memory; PS, processing speed; GSFS, Global Social Functioning Score.

In the HC group, 36% of executive functioning variance could be explained by leukocyte activity (WBC-AL) alone or in combination with PLT (41.8%). The significant combinations of leukocyte (WBC-AL, -M, and -AM), anti-inflammatory (IL-10), and pro-inflammatory activity (TNF-α) together with oxidative stress biomarkers (GSH, mROS, and SOD) explained 39.5–69.2% of the executive functioning variance at TY. Moreover, leukocyte (WBC-M) and pro-inflammatory activity (CRP) were considered significant to predict social functioning, explaining 39% of the variance at TY (Figure 3).

## Discussion

To the best of our knowledge, this is the first study to evaluate the associations between peripheral immune–inflammatory biomarkers and cognition and social functioning across chronic somatic and mental illnesses from a transdiagnostic perspective.

The participants of our cohort with SMIs demonstrated worse social and executive functioning compared to those participants who were healthy and diabetic as in the previous studies. In particular, impaired cognitive flexibility and processing speed registered across those with SMIs when compared with HCs and people with T2DM. Interestingly, IL-6 alone predicted 39.8 and 13.8% of cognitive flexibility in MDD and SZ respectively and 33.7% of processing speed in participants with MDD. Furthermore, the different combinations of cytokines, oxidative stress biomarkers, and cell populations predicted up to 46–57.3% of the ED among SMIs, a greater percentage than for HCs (36–41.8%); and IL-6 alone anticipated 33.2% of the ED score in MDD. Indeed, IL-6 has been long recognized as a consistent marker of systemic inflammation and stress, which may reflect hypothalamic–pituitary–adrenal axis overactivation. Furthermore, in those with MDD, its levels fluctuate depending on the disease stage and tend to normalize during remission states (63).

Like fatigue or anhedonia, neurocognitive impairment is a clinical trait of psychiatric disorders and several chronic somatic diseases, such as DM and coronary heart disease, which might be elicited by the background, low-grade inflammatory activity (2, 10, 64). This peripheral activation promotes central neuro-inflammation and, thus, modulation of neuronal signaling (7, 38), processes that may be more relevant in a certain subset of patients and could define, to some extent, a particular endophenotype among people with SMIs.

There is some evidence of an association between certain immune–inflammatory mediators and executive dysfunction in SMIs. In SZ, general cognitive function showed a negative correlation with IL-6, CRP, IL-1RA, and soluble TNF receptor 1 (sTNFR1). Similarly, the neurocognitive deficits in BD have been mostly related to CRP, IL-1RA, IL-6, and TNF-α. Even during remission, people with BD show impaired executive and visuospatial cognition, memory, and attention, all of which correlate with increased levels of serum TNF-α, IL-1, and IL-6 (22). Among them, the TNF superfamily is suspected to reflect neuroprogression, given that TNF-α concentration tends to rise in advanced stages of BD (5, 7, 11). In the case of MDD, TNF-α and its receptors, CRP and IL-6 have been the most related parameters (2, 11). Specifically, these three biomarkers have been broadly used to identify patients with depression, showing a baseline inflammation that may be clinically relevant (65), and 27% of patients with depression had a CRP concentration of more than 3 mg/dl (4). Moreover, CRP and IL-6 were able to predict impaired performance at a 12-year follow-up (18) for antidepressant treatment response (66).

Even under physiological conditions, peripheral and central immune–inflammatory mediators are required for the regulation of memory and learning processes according to the “cytokine model of cognitive function” (18). In diseases with an inflammatory basis, the return to homeostasis is hampered due to persistent immune–inflammatory activity (25, 63) and this sustained dysregulation may itself develop following David Barker's theory of “developmental programming” (10). Thus, chronic elevation of these mediators disrupts neural microenvironment balance, usually preserved by microglia, astrocytes and self-specific T-lymphocytes, and favors detrimental effects in brain regions like the hippocampus (67). Overactive microglia promote oxidative stress progression, brain-derived neurotrophic factor decrease, impairment of synaptic pruning and neuro-apoptosis, changes that contribute to alteration of cognitive, affective, and behavioral function (7). Aberrant stimulation of indoleamine 2,3-dioxygenase and tryptophan catabolites formation may be a convergent phenomenon of SMIs, cardiometabolic disease, and cancer with distinct pathophysiological roles (68, 69). In this context, differential tryptophan metabolism depending on astrocyte or microglia predominance determines the balance between tryptophan catabolites, such as neuroprotective kynurenic acid and pro-inflammatory quinolinic acid (27). Some implications of kynurenine pathway induction are serotonin depletion and modulation of glutamatergic and dopaminergic neurotransmission (70). Regarding executive function more specifically, *N*-methyl-d-aspartate (NMDA) receptor-mediated glutamatergic activity plays a central role in memory, synaptic plasticity, and neuronal development, while its overstimulation is implied in neuronal death (71).

The systemic immune–inflammatory activity observed in SMIs could be a trigger or a consequence of these diseases, or even an attempt to compensate for deleterious cellular events (72). The dynamic between the immune–inflammatory response and the novel compensatory immune-regulatory reflex systems is currently an area gaining attention in SZ, mood, and other neuroimmune disorders (73–75).

Based on the current evidence, several research groups have designed anti-inflammatory add-on therapies. Preclinical data support that certain nutrients, such as omega-3 polyunsaturated fatty acids, polyphenols, and folate, can have positive effects on the bidirectional “gut–brain axis” and some hygienic interventions, comprising strategies based on nutrition and physical exercise, have been designed. However, robust clinical trial support is still missing (76, 77). Several pharmacological interventions, mainly using *N*-acetylcysteine (NAC), non-steroidal anti-inflammatory drugs (NSAIDs), celecoxib, and anti-cytokines (31, 33, 66), have shown positive effects on SMI symptoms and clinical improvement was more significant among patients with higher baseline inflammatory states. Although subthreshold peripheral inflammatory states have been identified as contributors of cognitive impairment in SZ and BD, there is a flagrant lack of reports of the responses of neurocognitive deficits to the pharmacological interventions (11). Some preliminary data from celecoxib and mouse models of neurotoxicity and Parkinson's disease treated with NSAIDs show benefits for cognitive domains (18). Similarly, our results support the hypothesis that at least a part of the executive impairment observed in certain chronic diseases could be explained by altered immune–inflammatory activity and, therefore, improve with immune–inflammatory modulating strategies.

### Limitations

Several limitations of the present study must be considered. Our sample size was not very large (*n* = 165) and 40 patients were lost during follow-up. This hinders drawing conclusions for the general population. Furthermore, the studied phenomenon theoretically affected a limited subgroup of patients with SMIs and thus the sample size determined the observations to some extent. The participants' treatment also as usual was continued without no specific interventions; thus, it was difficult to anticipate substantial changes related to psychopharmacological pattern. Therefore, the studies with similar aims would benefit from cluster analysis and larger sample populations, which would supposed to take the psychopharmacologic pattern into account. Moreover, the inflammatory processes among the participants with MDD, SZ, and BD fluctuated depending on the status of the disorder (stable, relapse, remission) and pharmacological interventions. Thus, detectable inflammatory markers very likely varied because our participants had already been diagnosed, were at different disease stages, and were only followed-up for 1 year. The assessment of social functioning was performed by means of self-report questionnaires, so the scores could have been biased by the person's introspection and memory capacities; thus, constituting another limitation in this study.

## Conclusions and Future Directions

The multifactorial character of chronic somatic and mental disorders hinders the achievement of integral management for entities, such as T2DM and SMIs. However, the fact that these illnesses show overlapping clinical and biological traits opens the possibility to explore transdiagnostic links, such as immune–inflammatory mechanisms, to design new management strategies. Although the background inflammatory activity needs to be further defined, it has an unequivocal association with the cognitive and social functioning of these patients (10, 27, 66, 78) and accounts for a prominent part of the burden attributed to these diseases, in the short and long term (3).

Our current results elucidated the predictive power of peripheral biomarkers for immune–inflammatory activity in relation to social and executive functioning in patients with SMIs and T2DM. In this complex interrelation between the neuroendocrine and the immune–inflammatory processes, the presence of a transdiagnostic convergence across somatic and mental disorders of impaired inflammatory activity reinforces the significance of the scientific knowledge regarding comorbidity (79, 80) as an essential field of research with a holistic perspective. The studies investigating comorbidity and its overlapping pathways have already shown that these associations provide an invaluable chance to broaden the horizons of what is currently known about the pathogenesis, progress, and repercussions of these pathologies, and may allow further progress in the context of “precision psychiatry” paradigm (81).

To delve into the shared mechanisms and to reduce heterogeneity among patients with SMIs (82), a task for the future is to design studies in which psychiatric patients are followed from disease onset, including the treatment approach and a precisely described disease progression. Additionally, the exploration of pathophysiological-related entities could enrich the present perspectives and enhance our understanding of the underlying mechanism, taking, for instance, NMDA-R encephalitis as a valid comparative model for SZ.

With all this evidence, future research should doubtlessly further deconstruct psychiatric disorders. The cluster determination would help account for the heterogeneity that is currently found across individuals with SMIs to guarantee not just symptomatic improvement, but also an actual reintegration of patients, for whom the improvement of cognitive and social functions is essential. Thus, the characterization of endophenotypes among patients, in which immune–inflammatory activity could have a central and deciding role, may offer potential therapeutic and clinical interventions in the future.

## Data Availability Statement

The original contributions presented in the study are included in the article/Supplementary Material, further inquiries can be directed to the corresponding author/s.

## Ethics Statement

The studies involving human participants were reviewed and approved by Comité de Ética del Hospital Clinic-Universitari de Valencia. The patients/participants provided their written informed consent to participate in this study.

## Author Contributions

MG-C, JS-O, PC-G, VB-M, and RT-S: conception and design of the study, acquisition and analysis of data, and drafting the manuscript and figures. GS-V, CS-M, VV, IE-L, AH-M, JV-L, EV, and JCL: drafting the manuscript and figures. JV-F and RM-B: formal analysis. All authors have read and accepted the published version of the manuscript.

## Funding

This work was supported by the Carlos III Health Institute (ISCIII) (Grant Number PI19/0838), the European Regional Development Fund, and the Ministry of Education of the Valencian Regional Government (Grant Number PROMETEO/2019/027).

## Conflict of Interest

EV has received grants and served as consultant, advisor or CME speaker for the following entities: AB-Biotics, Abbott, AbbVie, Angelini, Boehringer–Ingelheim, Dainippon Sumitomo Pharma, Ferrer, Gedeon Richter, GH Research, Janssen, Lundbeck, Novartis, Otsuka, Sage, Sanofi–Aventis, Sunovion, and Takeda, outside the submitted work. The remaining authors declare that the research was conducted in the absence of any commercial or financial relationships that could be construed as a potential conflict of interest.

## Publisher's Note

All claims expressed in this article are solely those of the authors and do not necessarily represent those of their affiliated organizations, or those of the publisher, the editors and the reviewers. Any product that may be evaluated in this article, or claim that may be made by its manufacturer, is not guaranteed or endorsed by the publisher.
