# Supplementary material for: Immune–Inflammatory Biomarkers Predict Cognition and Social Functioning in Patients With Type 2 Diabetes Mellitus, Major Depressive Disorder, Bipolar Disorder, and Schizophrenia: A 1-Year Follow-Up Study
Source: Front Neurol. 2022 Jun 2;13:883927. doi: 10.3389/fneur.2022.883927 (PMC9201031; doi:10.3389/fneur.2022.883927)
Supplement: Supplementary file 1 [file Data_Sheet_1.DOCX]

| **Table S1. Predictive biomarkers at T1 of executive performance and social functioning at T2 to HC** | | | | | |
| --- | --- | --- | --- | --- | --- |
| **Dependent variables at T2** | **Predictors at T1 associated** | ***β*** | **95% CI** | ***t*** | **Percent of variance explained (adjusted *R^2^*)** |
| ***Executive functions*** | | | | | |
| **CF** | **mROS** | -0.70 | -0.01 to 0.00 | 2.82** | 62.5 |
|  | **SOD** | 0.78 | 0.00 to 0.02 | 3.19** |  |
|  | **WBC-AL** | -0.75 | -1.20 to -0.43 | 4.52**** |  |
| **VF** | **TNF-α** | -0.44 | -0.44 to -0.01 | 2.25* | 39.5 |
|  | **GSH** | 0.49 | 0.001 to 0.007 | 2.52* |  |
| **WM** | **IL-10** | -0.32 | -0.0004 to 0.00 | 1.87* | 69.2 |
|  | **mROS** | -0.69 | -0.01 to 0.00 | 3.89** |  |
|  | **PLT** | 0.68 | 0.00 to 0.01 | 3.60** |  |
|  | **WBC-M** | 0.59 | 0.05 to 0.31 | 3.09** |  |
|  | **WBC-AM** | -0.71 | -3.57 to -0.92 | 3.67** |  |
| **PS** | **TNF-α** | -0.35 | -0.24 to 0.01 | 1.83* | 62.7 |
|  | **PLT** | 0.54 | 0.001 to 0.009 | 2.69** |  |
|  | **WBC-AL** | -0.45 | -0.60 to -0.07 | 2.75** |  |
|  | **WBC-M** | 0.56 | 0.04 to 0.23 | 3.26** |  |
| ***Executive domain*** | | | | | |
| **ED** | **WBC-AL** | -0.60 | -0.72 to -0.13 | 3.09** | 36.0 |
|  | **PLT** | 0.51 | 0.000 to 0.009 | 2.33* | 41.8 |
|  | **WBC** | -0.73 | -0.21 to -0.04 | 3.31** |  |
| ***Social functioning*** | | | | | |
| **GSFS** | **PCR-us** | 0.37 | -0.02 to 0.36 | 1.98* | 39.0 |
|  | **WBC-M** | 0.49 | 0.02 to 0.32 | 2.53* |  |
| Abbreviations: T1 = Time 1, T2 = Time 2, HC = Healthy Control, CF = Cognitive Flexibility, VF = Verbal Fluency, WM = Working Memory, PS = Processing Speed, ED = Executive Domain, GSFS = Global Social Functioning Score, IL-10 = Interleukin-10, TNF-α = Tumor Necrosis Factor alpha, PCR-us = Ultra-sensitive Protein C, GSH = Glutathione, mROS = Mitochondrial Reactive Oxygen Species, SOD = Superoxide Dismutase, PLT = Blood Platelets, WBC = White Blood Cell, AL = Absolute Lymphocytes, M = Monocytes, AM = Absolute Monocytes. (*p ≤ 0.05; **p ≤ 0.01; ***p ≤ 0.001; ****p ≤ 0.0001). | | | | | |

| **Table S2. Predictive biomarkers at T1 of executive performance and social functioning at T2 to T2DM** | | | | | |
| --- | --- | --- | --- | --- | --- |
| **Dependent variables at T2** | **Predictors at T1 associated** | ***β*** | **95% CI** | ***t*** | **Percent of variance explained (adjusted *R^2^*)** |
| ***Executive functions*** | | | | | |
| **CF** | **PCR-us** | -0.42 | -0.13 to -0.01 | 2.48* | 38.6 |
|  | **GSH** | 0.53 | 0.00 to 0.01 | 3.15** |  |
| **VF** | **IL-6** | -0.48 | -0.62 to -0.07 | 2.66** | 23.5 |
| **WM** | **GSH** | 0.44 | 0.00 to 0.01 | 2.06* | 32.5 |
|  | **ROS** | -0.39 | -0.07 to 0.00 | 1.90* |  |
|  | **HGB** | 1.92 | 0.54 to 4.50 | 2.65** |  |
|  | **HCT** | -1.87 | -1.40 to -0.12 | 2.49* |  |
| **PS** | **GSH** | 0.45 | 0.00 to 0.01 | 2.21* | 25.9 |
|  | **HGB** | 1.42 | -0.03 to 2.62 | 2.02* |  |
|  | **HCT** | -1.35 | -0.80 to 0.03 | 1.89* |  |
| ***Executive domain*** | | | | | |
| **ED** | **PCR-us** | -0.37 | -0.11 to 0.00 | 1.99* | 35.8 |
|  | **GSH** | 0.52 | 0.00 to 0.01 | 2.63** |  |
|  | **HGB** | 1.43 | 0.02 to 2.47 | 2.12* |  |
|  | **HCT** | -1.41 | -0.77 to 0.00 | 2.03* |  |
| ***Social functioning*** | | | | | |
| **GSFS** | **GSH** | 0.42 | 0.00 to 0.01 | 2.24* | 28.3 |
|  | **SOD** | -0.41 | -0.08 to 0.00 | -2.17* |  |
| Abbreviations: T1 = Time 1, T2 = Time 2, T2DM = Type-2 Diabetes Mellitus, CF = Cognitive Flexibility, VF = Verbal Fluency, WM = Working Memory, PS = Processing Speed, ED = Executive Domain, GSFS = Global Social Functioning Score, IL-6 = Interleukin-6, PCR-us = Ultra-sensitive Protein C, GSH = Glutathione, ROS = Reactive Oxygen Species, SOD = Superoxide Dismutase, HGB = Hemoglobin, HCT = Hematocrit. (*p ≤ 0.05; **p ≤ 0.01; ***p ≤ 0.001; ****p ≤ 0.0001). | | | | | |

| **Table S3. Predictive biomarkers at T1 of executive performance and social functioning at T2 to MDD** | | | | | |
| --- | --- | --- | --- | --- | --- |
| **Dependent variables at T2** | **Predictors at T1 associated** | ***β*** | **95% CI** | ***t*** | **Percent of variance explained (adjusted *R^2^*)** |
| ***Executive functions*** | | | | | |
| **CF** | **IL-6** | -0.63 | -0.88 to -0.27 | 3.90*** | 39.8 |
|  | **IL-10** | -0.31 | -0.02 to 0.00 | 1.89* | 51.7 |
|  | **TNF-α** | -0.34 | -0.29 to 0.00 | 2.14* |  |
|  | **PCR-us** | -0.35 | -0.18 to 0.00 | 2.19* |  |
|  | **ROS** | 0.33 | 0.00 to 0.008 | 2.09* |  |
| **VF** | **SOD** | 0.33 | 0.00 to 0.03 | 1.87* | 42.7 |
|  | **WBC** | -4.65 | -3.89 to -0.66 | 2.94** |  |
|  | **WBC-N** | -2.45 | -0.49 to -0.11 | 3.28** |  |
|  | **WBC-AN** | 5.09 | 1.10 to 6.40 | 2.95** |  |
| **WM** | **IL-6** | -0.72 | -0.89 to -0.26 | 3.84*** | 45.1 |
|  | **IL-10** | 0.65 | 0.00 to 0.04 | 3.23** |  |
|  | **SOD** | 0.39 | 0.00 to 0.04 | 2.13* |  |
| **PS** | **IL-6** | -0.58 | -0.58 to -0.14 | 3.42** | 33.7 |
|  | **PCR-us** | -0.43 | -0.14 to -0.02 | 2.83** | 56.3 |
|  | **WBC** | -4.79 | -3.79 to -0.96 | 3.50** |  |
|  | **WBC-N** | -2.43 | -0.47 to -0.13 | 3.74*** |  |
|  | **WBC-AN** | 5.18 | 1.53 to 6.19 | 3.45** |  |
| ***Executive domain*** | | | | | |
| **ED** | **IL-6** | -0.57 | -0.59 to -0.14 | 3.38** | 33.2 |
|  | **PCR-us** | -0.35 | -0.12 to 0.00 | 2.27* | 57.3 |
|  | **WBC** | -4.50 | -3.70 to -0.79 | 3.24** |  |
|  | **WBC-N** | -2.50 | -0.49 to -0.14 | 3.77*** |  |
|  | **WBC-AN** | 4.92 | 1.30 to 6.09 | 3.28** |  |
|  | **WBC-M** | -0.32 | -0.09 to 0.00 | 1.97* |  |
| ***Social functioning*** | | | | | |
| **GSFS** | **HGB** | -0.39 | -2.44 to -0.01 | -2.10* | 23.8 |
|  | **WBC-AN** | -0.35 | -1.72 to 0.08 | -1.87* |  |
| Abbreviations: T1 = Time 1, T2 = Time 2, MDD = Mayor Depressive Disorder, CF = Cognitive Flexibility, VF = Verbal Fluency, WM = Working Memory, PS = Processing Speed, ED = Executive Domain, GSFS = Global Social Functioning Score, IL-6 = Interleukin-6, IL-10 = Interleukin-10, TNF-α = Tumor Necrosis Factor alpha, PCR-us = Ultra-sensitive Protein C, ROS = Reactive Oxygen Species, SOD = Superoxide Dismutase, HGB = Hemoglobin, WBC = White Blood Cell, N = Neutrophils, AN = Absolute Neutrophils, M = Monocytes. (*p ≤ 0.05; **p ≤ 0.01; ***p ≤ 0.001; ****p ≤ 0.0001). | | | | | |

| **Table S4. Predictive biomarkers at T1 of executive performance and social functioning outcomes at T2 to BD** | | | | | |
| --- | --- | --- | --- | --- | --- |
| **Dependent variables at T2** | **Predictors at T1 associated** | ***β*** | **95% CI** | ***t*** | **Percent of variance explained (adjusted *R^2^*)** |
| ***Executive functions*** | | | | | |
| **CF** | **HGB** | 1.53 | 0.54 to 3.14 | 2.91** | 26.4 |
|  | **HCT** | -1.29 | -0.98 to -0.08 | 2.46* |  |
| **VF** | **IL-6** | 0.51 | 0.02 to 0.24 | 2.54* | 49.8 |
|  | **PCR-us** | -0.53 | -0.30 to -0.03 | 2.58* |  |
|  | **SOD** | 0.44 | 0.002 to 0.009 | 2.96** |  |
|  | **HGB** | 1.17 | 0.14 to 1.46 | 2.52* |  |
|  | **HCT** | -0.91 | -0.44 to 0.01 | 1.93* |  |
| **WM** | **WBC-N** | 3.28 | 0.39 to 1.15 | 4.21**** | 54.4 |
|  | **WBC-L** | 2.28 | 0.10 to 1.03 | 2.55* |  |
|  | **WBC-AL** | 0.83 | -0.10 to 6.49 | 2.00* |  |
|  | **WBC-M** | 1.38 | 0.43 to 2.59 | 2.91** |  |
|  | **WBC-AM** | -1.31 | -24.9 to -0.62 | 2.17* |  |
| **PS** | **PCR-us** | -0.34 | -0.26 to 0.00 | 2.04* | 29.9 |
|  | **GSH** | 0.46 | 0.00 to 0.01 | 2.18* |  |
|  | **SOD** | 0.47 | 0.00 to 0.01 | 2.19* |  |
| ***Executive domain*** | | | | | |
| **ED** | **WBC** | -9.41 | -7.61 to -2.06 | 3.61*** | 48.0 |
|  | **WBC-AN** | 10.63 | 2.90 to 9.55 | 3.87*** |  |
|  | **WBC-AL** | 3.09 | 3.51 to 10.12 | 4.26**** |  |
|  | **WBC-M** | 2.15 | 0.36 to 2.34 | 2.82** |  |
|  | **WBC-AM** | -2.24 | 23.08 to -2.07 | 2.47* |  |
| ***Social functioning*** | | | | | |
| **GSFS** | **TNF-α** | -0.36 | -0.25 to 0.00 | -2.07* | 24.7 |
|  | **ROS** | -0.31 | -0.02 to 0.00 | -1.87* |  |
|  | **WBC-L** | -0.35 | -0.11 to 0.00 | -1.93* |  |
| Abbreviations: T1 = Time 1, T2 = Time 2, BD = Bipolar Disorder, CF = Cognitive Flexibility, VF = Verbal Fluency, WM = Working Memory, PS = Processing Speed, ED = Executive Domain, GSFS = Global Social Functioning Score, IL-6 = Interleukin-6, TNF-α = Tumor Necrosis Factor alpha, PCR-us = Ultra-sensitive Protein C, GSH = Glutathione, ROS = Reactive Oxygen Species, SOD = Superoxide Dismutase, HGB = Hemoglobin, HCT = Hematocrit, WBC = White Blood Cell, N = Neutrophils, AN = Absolute Neutrophils, L = Lymphocytes, AL = Absolute Lymphocytes, M = Monocytes, AM = Absolute Monocytes. (*p ≤ 0.05; **p ≤ 0.01; ***p ≤ 0.001; ****p ≤ 0.0001). | | | | | |

| **Table S5. Predictive biomarkers at T1 of cognitive and social outcomes at T2 to SZ** | | | | | |
| --- | --- | --- | --- | --- | --- |
| **Dependent variables at T2** | **Predictors at T1 associated** | ***β*** | **95% CI** | ***t*** | **Percent of variance explained (adjusted *R^2^*)** |
| ***Executive functions*** | | | | | |
| **CF** | **IL-6** | -0.37 | -0.67 to 0.01 | 1.99* | 13.8 |
| **VF** | **PCR-us** | -0.42 | -0.10 to -0.01 | 2.59** | 39.5 |
|  | **mROS** | -0.35 | -0.01 to 0.00 | 2.01* |  |
|  | **SOD** | 0.35 | 0.00 to 0.02 | 1.98* |  |
| **WM** | **PCR-us** | -0.40 | -0.29 o -0.01 | 2.21* | 16.4 |
|  | **mROS** | -0.75 | -0.05 to -0.01 | 3.41** | 45.7 |
|  | **SOD** | 0.60 | 0.02 to 0.10 | 3.18** |  |
|  | **WBC** | -3.35 | -5.79 to -1.12 | 3.08** |  |
|  | **WBC-M** | -2.36 | -5.66 to -1.12 | 3.11** |  |
|  | **WBC-AM** | 3.41 | 17.42 to 82.20 | 3.19** |  |
| **PS** | **mROS** | -0.77 | -0.03 to 0.00 | 3.44** | 42.3 |
|  | **SOD** | 0.59 | 0.01 to 0.06 | 3.05** |  |
|  | **WBC** | -2.87 | -3.23 to -0.33 | 2.55* |  |
|  | **WBC-M** | -1.98 | -3.12 to -0.30 | 2.53* |  |
|  | **WBC-AM** | 2.96 | 5.92 to 46.19 | 2.69* |  |
| ***Executive domain*** | | | | | |
| **ED** | **mROS** | -0.80 | -0.03 to 0.00 | 3.65*** | 46.0 |
|  | **SOD** | 0.64 | 0.01 to 0.06 | 3.38** |  |
|  | **WBC** | -3.22 | -3.43 to -0.60 | 2.96** |  |
|  | **WBC-M** | -2.13 | -3.23 to -0.48 | 2.80** |  |
|  | **WBC-AM** | 3.25 | 9.20 to 48.51 | 3.05** |  |
| ***Social functioning*** | | | | | |
| **GSFS** | **IL-6** | 0.40 | -0.01 to 0.56 | 1.95* | 35.7 |
|  | **IL-10** | 0.37 | 0.00 to 0.01 | 1.89* |  |
|  | **PCR-us** | -0.32 | -0.15 to 0.01 | -1.83* |  |
|  | **ROS** | -0.40 | -0.01 to 0.00 | -2.07* |  |
|  | **mROS** | -0.44 | -0.02 to 0.00 | -2.30* |  |
| Abbreviations: T1 = Time 1, T2 = Time 2, SZ = Schizophrenia, CF = Cognitive Flexibility, VF = Verbal Fluency, WM = Working Memory, PS = Processing Speed, ED = Executive Domain, GSFS = Global Social Functioning Score, IL-6 = Interleukin-6, IL-10 = Interleukin-10, PCR-us = Ultra-sensitive Protein C, ROS = Reactive Oxygen Species, mROS = Mitochondrial Reactive Oxygen Species, SOD = Superoxide Dismutase, WBC = White Blood Cell, M = Monocytes, AM = Absolute Monocytes. (*p ≤ 0.05; **p ≤ 0.01; ***p ≤ 0.001; ****p ≤ 0.0001). | | | | | |
